# Experienced physicians benefit from analyzing initial diagnostic hypotheses

**Published:** 2013-03-31

**Authors:** Adam Bass, Colin Geddes, Bruce Wright, Sylvain Coderre, Remy Rikers, Kevin McLaughlin

**Affiliations:** 1 University of Calgary, Calgary, Alberta, Canada; 2 Glasgow University, Glasgow, Scotland, UK; 3 Erasmus University, Rotterdam, the Netherlands

## Abstract

**Background:**

Most incorrect diagnoses involve at least one cognitive error, of which premature closure is the most prevalent. While metacognitive strategies can mitigate premature closure in inexperienced learners, these are rarely studied in experienced physicians. Our objective here was to evaluate the effect of analytic information processing on diagnostic performance of nephrologists and nephrology residents.

**Methods:**

We asked nine nephrologists and six nephrology residents at the University of Calgary and Glasgow University to diagnose ten nephrology cases. We provided presenting features along with contextual information, after which we asked for an initial diagnosis. We then primed participants to use either hypothetico-deductive reasoning or scheme-inductive reasoning to analyze the remaining case data and generate a final diagnosis.

**Results:**

After analyzing initial hypotheses, both nephrologists and residents improved the accuracy of final diagnoses (31.1% vs. 65.6%, *p* < 0.001, and 40.0% vs. 70.0%, *p* < 0.001, respectively). We found a significant interaction between experience and analytic processing strategy (*p* = 0.02): nephrology residents had significantly increased odds of diagnostic success when using scheme-inductive reasoning (odds ratio [95% confidence interval] 5.69 [1.59, 20.33], *p* = 0.07), whereas the performance of experienced nephrologists did not differ between strategies (odds ratio 0.57 [0.23, 1.39], *p* = 0.20).

**Discussion:**

Experienced nephrologists and nephrology residents can improve their performance by analyzing initial diagnostic hypotheses. The explanation of the interaction between experience and the effect of different reasoning strategies is unclear, but may relate to preferences in reasoning strategy, or the changes in knowledge structure with experience.

## Introduction

Despite ongoing improvements in physician training and healthcare delivery, 10 – 15% of patients are still misdiagnosed.^1^ While diagnostic errors often have multiple etiologies, faulty cognition is implicated in approximately three quarters of all errors.^2^ So why are cognitive errors so frequent?

Diagnosing, and decision-making in general, involves two cognitive processes.^3^–^5^ The first is automatic information processing, also referred to as intuition or pattern recognition. This requires minimal use of working memory, and involves rapid, subconscious processing of data that are largely contextual, to reach a single diagnosis or a short list of possible diagnoses. By contrast, analytic information processing involves conscious evaluation of case data and potential diagnoses by working memory until the best fitting diagnosis is selected. Diagnostic error may result from faults in either, or both, of these processes, but the most frequent explanation is “premature closure”, where a final diagnosis is selected before sufficient information has been processed.^1^

If insufficient analytic processing causes diagnostic error then further analysis of diagnostic hypotheses might improve performance.^6^ This metacognitive approach is supported by several studies demonstrating mitigation of premature closure and improved accuracy of final diagnoses.^7^–^11^ But further analysis does not always improve decision making, and the psychology and medical education literature is replete with studies demonstrating that analysis can result in poorer diagnoses/decisions.^12^–^14^ This equipoise suggests that the effect of analytic processing on diagnostic performance may be modified by other variables, such as the structure and complexity of the content area being studied, experience within this content area, and perhaps individual preferences of problem solving strategies.^5^

These potential effect modifiers are not independent. Complexity is inversely related to experience, and problem-solving preferences change with experience. Novice learners solve problems by first creating mental models from their underlying knowledge and perception of the problem, and then analyze each model until the best fit is selected.^15^,^16^ When studying problem-solving in medicine we typically refer to “models” as “diagnostic hypotheses”, and replace “model-based reasoning” with “hypothetico-deductive reasoning”. With experience, this form of reasoning becomes increasingly complex as improved knowledge allows for a larger number of more elaborate models to be generated, thus increasing cognitive load.^15^,^17^ To counter this, more experienced learners abstract domain-specific rules and then form rule-based schemas to allow them to analyze problems without building models, or by building fewer models.^18^–^20^ In theory, adopting “rule-based reasoning” (or “scheme-inductive reasoning”) allows for more efficient diagnosing. Finally, with further experience the role of analytic processing is diminished and diagnoses are increasingly made by automatic processing alone.^21^ Experienced physicians may be afforded the luxury of diagnosing without analysis due to improved perception and ability to generate accurate initial hypotheses, and/or because they have developed mental shortcuts, or “heuristics”, whereby they omit or obscure the intermediary steps of analytic processing.^22^,^23^

Experienced physicians develop heuristics as these are more likely to help than hinder diagnostic reasoning.^24^ Yet with increasing experience and reliance on automatic processing and heuristics, physicians are more susceptible to premature closure, are less willing to reconsider their diagnoses, and, consequently, become progressively error prone.^25^–^27^ If analyzing an initial hypothesis improves performance of less experienced learners, this may also work for experienced physicians. But we should not assume that metacognitive strategies are universally beneficial: if we force experienced physicians to abandon their preferred reasoning strategies and process information like novices they may perform like novices!^28^

In this study our objective was to evaluate the effect of analyzing an initial hypothesis on the diagnostic performance of practicing physicians. We predicted that if analysis is an effective metacognitive strategy then performance should improve with analysis. To test this hypothesis we manipulated the information processing strategies of two groups of physicians with differing degrees of experience – practicing nephrologists with ten or more years experience in caring for patients with kidney diseases, and nephrology subspecialty residents who had completed their training in internal medicine and were receiving further training in nephrology. After providing our participants with a brief clinical presentation and contextual information, we asked them to commit to an initial diagnosis – knowing that this would encourage the use of heuristics and risk premature closure. We then asked them to reconsider their diagnoses in light of further information on the case, and evaluated the impact of further analysis on diagnostic performance. Cognizant of the different analytic processing strategies, we primed our participants to use either hypothetico-deductive reasoning or scheme-inductive reasoning, thus allowing us to compare the impact of each strategy on diagnostic performance.

## Methods

Our study participants were nine practicing nephrologists with more than ten years of clinical experience in the care of nephrology patients, and six nephrology subspecialty residents at the University of Calgary or Glasgow University. The nephrology subspecialty residents had completed at least three years of training in internal medicine and were enrolled in nephrology subspecialty training programs of two year duration. The Conjoint Medical and Research Ethics Board at the University of Calgary granted ethical approval for the study and each participant provided informed consent prior to entry into the study.

**Figure 1:**
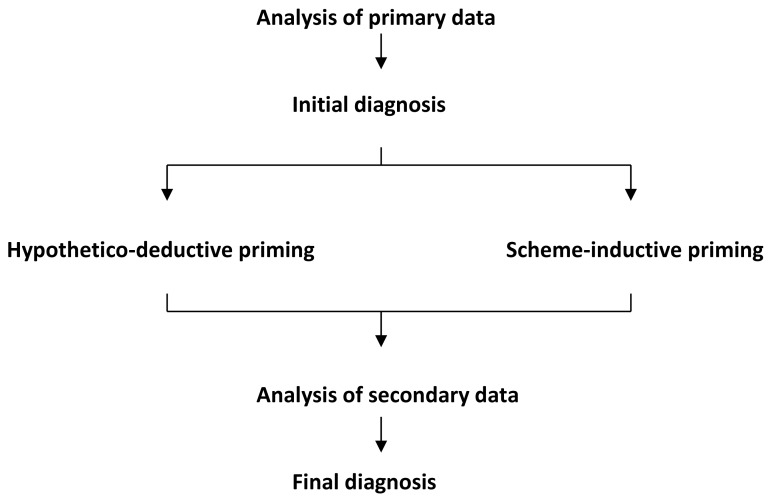
Overview of the study design.

We randomized our participants to receive one of two booklets containing ten identical nephrology cases in the same order. Each case began with primary data that included the presenting complaint – which could be a symptom, sign, or abnormal test result – along with the patient’s age, gender, enabling conditions, and clinical setting. Based upon these data alone, we asked our participants to offer an initial diagnostic hypothesis. The booklets then differed in the reasoning instructions given for each case: if group 1 was given instructions to use hypothetico-deductive reasoning for case 1 then group 2 was instructed to use scheme-inductive reasoning (see descriptions of these instructions below). Using computer-generated random numbers, we randomized the sequence of reasoning instructions, and each group answered five questions using each reasoning strategy. Cognizant that restricting the time available for information processing might increase the risk of premature closure, we did not limit the time available to complete problems. Figure 1 provides an overview of our study design.

### Instructions for hypothetico-deductive reasoning

Instructions on hypothetico-deductive reasoning were similar to those used by Mamede et al.^10^ After offering an initial diagnostic hypothesis participants were asked to list their differential diagnoses. Following the presentation of secondary data, they were asked to consider, for each diagnosis, features consistent with this diagnosis, features inconsistent with this diagnosis, and features that would have been expected if this were the correct diagnosis. We then asked them to rank diagnoses and give their final diagnosis.

### Instructions for scheme-inductive reasoning

After offering an initial diagnostic hypothesis participants were asked to draw a diagnostic scheme that could be used to help diagnose the cause of this clinical presentation. We then asked them to use their diagnostic scheme to analyze secondary data and provide their final diagnosis.

### Evaluation of diagnostic performance

The cases used were based upon real patients. For each case the final diagnosis was either confirmed using the available gold standard, such as histology, or two experienced nephrologists (CG & KM) agreed upon a single best diagnosis. Participants’ diagnoses were considered correct if they matched, or were synonymous with, the agreed-upon final diagnosis.

### Statistical Analyses

We used Fisher’s exact test to compare the proportion of correct diagnoses for different degrees of clinical experience (experienced nephrologist vs. nephrology resident), and reasoning strategies. We used McNemar’s discordant pair analysis to compare the direction of change (incorrect to correct and vice versa) between initial and final diagnoses.

We used multiple logistic regression to evaluate the effect of reasoning strategy and clinical experience on diagnostic performance. In our regression model we also considered two-variable interaction terms, and used backward elimination to remove non-significant variables from the model. We used STATA version 11.0 for our statistical analyses.

## Results

### The effect of analysis on diagnostic performance

The mean accuracy of initial diagnostic hypotheses was 34.7%, and this did not differ between experienced nephrologists and nephrology residents (*p* = 0.30). After analysis of the initial hypothesis, 35% of diagnoses were changed. Of these, the vast majority resulted in an incorrect initial hypothesis being changed to a correct final diagnosis rather than a correct initial hypothesis being changed to an incorrect final diagnosis (96% vs. 4%, *p* < 0.0001). The likelihood of changing an initial diagnostic hypothesis did not differ between nephrologists and residents (*p* = 0.70), or the information processing strategy used (*p* = 0.90).

**Figure 2:**
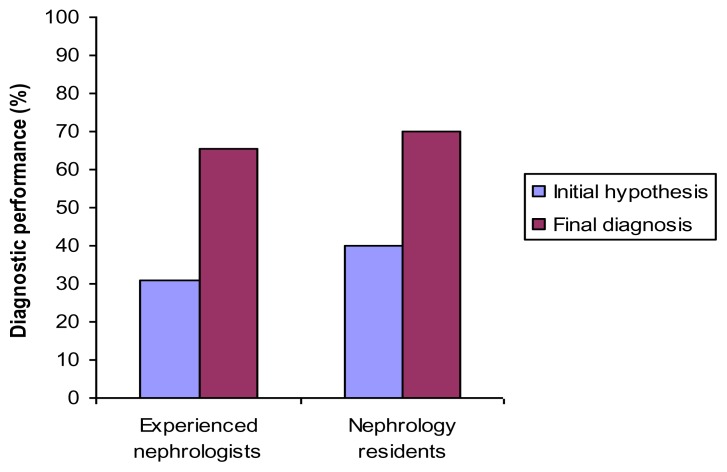
The effect on diagnostic performance of analyzing an initial hypothesis

After analyzing their initial diagnostic hypotheses, both nephrologists and residents improved the accuracy of their final diagnoses (31.1% vs. 65.6%, *p* <0.001, and 40.0% vs. 70.0%, *p* < 0.001, respectively). These data are shown in Figure 2.

### Hypothetico-deductive reasoning vs. scheme-inductive reasoning

**Figure 3:**
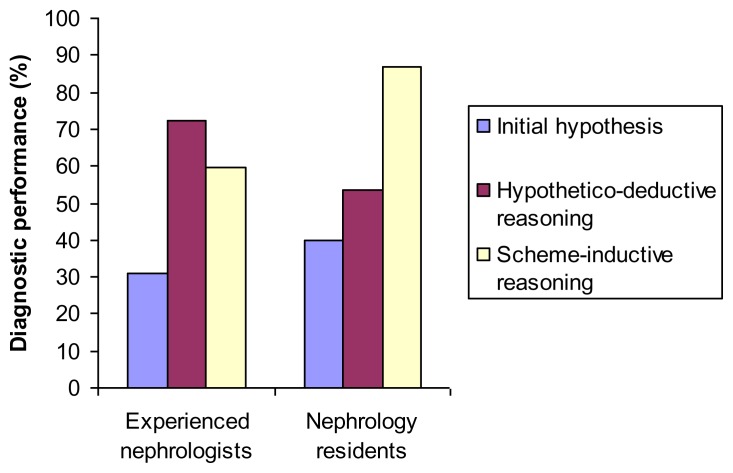
The effect on diagnostic performance of hypothetico-deductive reasoning vs. scheme-inductive reasoning

In our logistic regression model we found a significant interaction between clinical experience and the effect of different analytic processing strategies on diagnostic performance (*p* < 0.01). Stratifying our analysis by clinical experience, we found that nephrology residents had significantly increased odds of diagnostic success when using scheme-inductive reasoning as compared to hypothetico-deductive reasoning (odds ratio [95% confidence interval] 5.69 [1.59, 20.33], *p* < 0.01), whereas the performance of experienced nephrologists did not differ between strategies (odds ratio for scheme-inductive reasoning was 0.57 [0.23, 1.39], *p* = 0.20). Figure 3 shows the effect of the different analytic strategies on diagnostic performance.

When we analyzed compliance with priming instructions we found that our participants always followed the priming instructions for hypothetico-deductive reasoning, but experienced nephrologists failed to generate a diagnostic scheme (or described domain-specific rules) for 70% of the cases where they were asked to do so. By comparison, the non-compliance rate with scheme-inductive reasoning priming conditions among nephrology residents was only 3%. We therefore repeated our analyses based upon actual, rather than intended, analytical processing strategy used. In this post-hoc analysis the effect of analytic reasoning strategy on diagnostic performance was unchanged: nephrology residents had an increased odds of diagnostic success using scheme-inductive reasoning (odds ratio 5.15 [1.44, 18.34], *p* < 0.01), while there was no difference in the performance of experienced nephrologists (odds ratio 0.65 [0.20, 2.09], *p* = 0.50).

## Discussion

This study adds to the growing literature on the diagnostic benefits of analytic information processing. But rather than evaluating this approach in non-physicians or inexperienced trainees, our participants were physicians with considerable clinical experience – including those who are typically considered to be less flexible in their diagnostic reasoning strategies, and more prone to diagnostic error as a result of premature closure.^25^–^27^ The major finding in our study was that both experienced nephrologists and residents receiving subspecialty training in nephrology improved their performance after analyzing their initial diagnostic hypotheses. Upon analysis, they rarely substituted correct hypotheses with wrong diagnoses, but frequently rejected incorrect hypotheses in favour of correct diagnoses. Overall, the diagnostic success rate approximately doubled after analyzing their initial hypotheses.

The benefits of analytic processing are unlikely to be explained by more information processing simply being better rather than less. There are too many examples of performance declining with over-analysis to accept this explanation.^12^–^14^ In almost every field of study, experienced practitioners become progressively automated in their thoughts and actions (or “unconsciously competent”^29^), whereas analytic processing involves the use of working memory, where the logic of decisions can be examined, and their consequences anticipated. Studying experienced decision makers in many fields, Klein has concluded that experts – as distinct from simply experienced practitioners – use “recognition-primed decision making” where they consciously analyze the consequences of their initial decision, and then alter their decisions accordingly.^30^ Concurring with this, Epstein used the term “mindful practice” to describe decision making by expert diagnosticians.^31^ These descriptors imply that in the minds of experts cognitive processes complement each other rather than compete against each other, and that analysis is not better than intuition, or vice versa.^32^ In our study we did not try to force our participants to abandon their heuristics in favour of analysis. Rather, we actively encouraged them to use these heuristics to generate an early hypothesis – after which we provided an opportunity to debias their reasoning process by analyzing their initial hypothesis in light of further data. Thus, we forced our participants to use recognition-primed decision making, seemingly to good effect.

So, if it pays to analyze diagnostic hypotheses, is there a better way of doing this? Based upon observational studies suggesting that analytic processing naturally evolves from hypothetico-deductive to scheme-inductive reasoning, we predicted that scheme-inductive reasoning would be a more effective strategy.^18^–^20^ However, we found a significant interaction with experience: nephrology residents, but not experienced nephrologists, performed better when primed to use scheme-inductive reasoning.

The simple explanation for the interaction between experience and reasoning strategy is that residents prefer scheme-inductive reasoning, while experienced nephrologists prefer hypothetico-deductive reasoning. This is supported by the observation that most experienced nephrologists did not use scheme-inductive reasoning when primed to do so. Unfortunately our study was not designed to explain preferences in diagnostic reasoning, but we would speculate that each group prefers to do what they do on a daily basis. Nephrology residents, by virtue of their mandatory supervision, are forced to use analytic processing for each case they see in order to justify their diagnosis to the attending physician. They have sufficient training in nephrology to learn the domain-specific rules, and, in addition to a limited number of illness scripts, their knowledge structure contains abridged causal networks, replete with biomedical knowledge, within which domain-specific rules are stored.^33^,^34^ By comparison, experienced nephrologists are not forced to articulate the logic behind each diagnostic decision, and their knowledge structure is primarily composed of illness scripts, within which domain-specific rules have been encapsulated.^33^–^35^ Failure to use domain-specific rules on a daily basis may make them less accessible to experienced nephrologists, such that may be easier for them to recall previously encountered diagnoses, rather than rules.

Our study has some limitations. The small number of participants limited our power to detect differences within and between groups related to the use of different analytical reasoning strategies which resulted in imprecision and wide confidence intervals in our results. We only studied one clinical domain, so we cannot extrapolate the benefits of analytic processing to other domains. Although complex, nephrology problems are usually highly structured, and the domain-specific rules are well established – which may exaggerate the diagnostic benefits of analytic processing.^5^ We cannot assume that diagnosing in clinical domains where the rules are less clear, for example psychiatry, will also be enhanced by analytic processing. Although we observed a significant improvement in diagnostic performance after analytic processing, approximately one third of final diagnoses were incorrect. This was despite sufficient data being available on each case for the correct final diagnosis to be made. This suggests that other cognitive biases may have been operating – such as confirmation bias, where data supporting, rather than refuting, a diagnostic hypothesis, are over emphasized.^6^ More than thirty cognitive biases have been identified, so no single cognitive strategy is a panacea.^6^ There was no control group without a reasoning strategy and so we cannot comment on how to participants would have performed without a reasoning strategy. This was deliberate in our design since without specific instructions, there would have been no way of accurately determining which reasoning strategy or combination of strategies participants would have employed. Finally, it is unclear why experienced nephrologists failed to use schemes in our study. While there are a number of possible explanations for this finding, it is impossible for us to accurately determine why this is the case. Further studies are needed to identify and mitigate other cognitive biases.

### Implications for medical education

If the effect of a given information processing strategy on diagnostic performance may be modified by other variables, such as content area, experience, and individual preferences, making blanket recommendations on how to solve clinical problems is illogical. Recommendations on how to improve performance should target the cause(s) of poor performance, of which there are many. Our results suggest that for experienced physicians, as for novices, if the cognitive problem is premature closure, then analysis of initial hypotheses may be a cognitive solution. As to how these hypotheses should be analyzed, it appears that there is no consistently “better” analytic strategy, due to effect modification by other variables, such as clinical experience.

### Conclusion

We found that the diagnostic performance of experienced nephrologists and nephrology residents improved when they combined heuristics with analysis of their initial diagnostic hypotheses. The effectiveness of the analytical strategy used, scheme-inductive vs. hypothetico-deductive reasoning, varied with experience – likely due to divergent preferences for reasoning strategies, which may be related to differences in underlying knowledge structure. Although encouraging, our results also highlight that there are multiple sources of cognitive error, so one cognitive solution will not solve all cognitive problems.
